# ACSL4 serves as a novel prognostic biomarker correlated with immune infiltration in Cholangiocarcinoma

**DOI:** 10.1186/s12885-023-10903-5

**Published:** 2023-05-16

**Authors:** Shuochen Liu, Shilong Fan, Yirui Wang, Ruixiang Chen, Ziyi Wang, Yaodong Zhang, Wangjie Jiang, Yananlan Chen, Xiao Xu, Yue Yu, Changxian Li, Xiangcheng Li

**Affiliations:** 1 Hepatobiliary Center, The First Affiliated Hospital of Nanjing Medical University, Nanjing, China; 2 Key Laboratory of Liver Transplantation, Chinese Academy of Medical Sciences, Nanjing, China; 3 Department of Liver Surgery and Transplantation, Liver Cancer Institute and Zhongshan Hospital, Fudan University, Shanghai, China

**Keywords:** Cholangiocarcinoma, ACSL4, Prognosis, Immune infiltration, TCGA, GEO, Single-cell sequence

## Abstract

**Background:**

Cholangiocarcinoma (CHOL) is the second most common primary hepatic malignant tumor, following hepatocellular carcinoma (HCC). CHOL is highly aggressive and heterogeneous resulting in poor prognosis. The diagnosis and prognosis of CHOL has not improved in the past decade. Acyl-CoA synthetase long-chain family member 4 (ACSL4) is reported to be associated with tumors, however, its role in CHOL has not been revealed. This study is mainly for exploring the prognostic values and potential function of ACSL4 in CHOL.

**Methods:**

We investigated the expression level and prognostic value of ACSL4 in CHOL based on The Cancer Genome Atlas (TCGA) and Gene Expression Omnibus (GEO) datasets. TIMER2.0, TISIDB and CIBERSORT databases were utilized to assess the associations between ACSL4 and immune infiltration cells in CHOL. Single-cell sequencing data from GSE138709 was analyzed to study the expression of ACSL4 in different types of cells. ACSL4 co-expressed genes were analyzed by Linkedomics. Additionally, Western Blot, qPCR, EdU assay, CCK8 assay, transwell assay and wound healing assay were performed to further confirm the roles of ACSL4 in the pathogenesis of CHOL.

**Results:**

We found that the level of ACSL4 was higher in CHOL and it was correlated with the diagnosis and prognosis of CHOL patients. Then, we observed that the infiltration level of immune cells was related to the level of ACSL4 in CHOL. Moreover, ACSL4 and its co-expressed genes were mainly enriched in metabolism-related pathway and ACSL4 is also a key pro-ferroptosis gene in CHOL. Finally, knockdown of ACSL4 could reverse the tumor-promoting effect of ACSL4 in CHOL.

**Conclusions:**

The current findings demonstrated ACSL4 may as a novel biomarker for CHOL patients, which might regulate immune microenvironment and metabolism resulting in poor prognosis.

**Supplementary Information:**

The online version contains supplementary material available at 10.1186/s12885-023-10903-5.

## Introduction

Cholangiocarcinoma (CHOL) is a highly malignant tumor of the hepatobiliary system, which arises predominantly from the biliary epithelial cells [1]. The anatomic subtype of CHOL is classified into intrahepatic cholangiocarcinoma, perihilar cholangiocarcinoma and extrahepatic cholangiocarcinoma. Globally, CHOL is a relatively rare cancer, but its incidence and mortality have been increasing in recent decades. Unfortunately, CHOL is clinically silent in the early stage as symptoms only develop at advanced stage. The current non-invasive diagnosis of CHOL is not accurate enough [2]. Therefore, identifying reliable and specific biomarkers and therapeutic targets to distinguish CHOL and their molecular mechanisms is important.

Acyl-CoA synthetase long-chain family member 4 (ACSL4) is an enzyme that ligates polyunsaturated fatty acids (PUFAs) with coenzyme A (CoA) to PUFA-CoAs for their re-esterified in phospholipids by Lysophosphatidylcholine acyltransferase (LPCAT) enzymes [3]. ACSL4 is regarded as an essential gene in the progression of ferroptosis, especially in lipid composition [4, 5]. Meanwhile, higher ACSL4 expression in CHOL was observed by immunohistochemical staining compared with normal tissues [6]. Besides, ACSL4 also be found upregulated in hepatocellular carcinoma (HCC) [7] and colon cancer [8], which is related to increased aggressiveness and poor prognosis. But the function of ACSL4 in the pathogenesis of CHOL is still unclear.

CHOL is characterized by a prominent desmoplastic stroma with an inflammatory and immune cell infiltration [9, 10]. The tumor microenvironment, which includes stromal cells, innate and adaptive immune cells and the extracellular matrix [10], is heterogeneous and complex in CHOL and plays a functional component contributes to tumorigenesis. The degree of immune infiltration was associated prognosis [11], and we drew the same conclusion in our previous study [12]. However, the association between ACSL4 and immune infiltration in CHOL is still unclear.

Ferroptosis is an iron-dependent form of cell death, and is distinct from autophagy, necrosis and apoptosis in morphology, genetics, and biochemistry [13]. Ferroptosis could be a novel target to improve the sensitivity of chemotherapy, radiotherapy and immunotherapy. In a previous study, we constructed a 5 genes ferroptosis-related signature, including ACSL3, ACLS4, MUC1, SLC7A11 and SLC38A1. We found high ACSL4 or MUC1 expression in patients indicated poor overall survival, while the other 3 genes have no significant prognosis value. MUC1 is related to prognosis in CHOL [14], but the relationship between ACSL4 and prognosis in CHOL is unknown. Therefore, in the present study, we choose ACSL4 to further explore its function and correlation with ferroptosis in CHOL.

In this study, the ACSL4 expression, prognostic value, immune infiltration and co-expressed gene network in CHOL patients were investigated based on RNA-seq and scRNA-seq from TCGA and GEO databases, which were analyzed by R software package and online databases. We combined TCGA-CHOL and GSE107943 datasets into a meta dataset by removing batch effect and do further studies in this larger sample size dataset. In addition, we also verified the result in our CHOL samples by qPCR and Western Blot. We confirm, for the first time, that ACSL4 is a tumor promoter in CHOL in *vitro* by Knockdown of ACSL4 in CHOL cell lines. Moreover, we found ACSL4 was crucial in ferroptosis in CHOL. The results suggested that ACSL4 could promote CHOL and may be a prognostic biomarker and potential target for the treatment of CHOL.

## Materials and methods

### Dataset analyses

The RNA expression data and clinical information were retrieved from the Gene Expression Omnibus (GEO) database (https://www.ncbi.nlm.nih.gov/geo) (GSE76297 [15] and GSE107943 [16]) and The Cancer Genome Atlas (TCGA) database (https://www.cancer.gov/tcga). The data acquired from the TCGA and GEO database were transferred to Transcripts Per Kilobase Million (TPM) for further analysis.

The single-cell sequencing data of 8 samples from 5 patients with intrahepatic cholangiocarcinoma was acquired from GEO database (GSE138709 [17]). Seurat R package was used to analyze raw data from GSE138709 in R with R studio [18]. Cells were excluded in accordance with following criteria. (1) more than 20% mitochondria-related genes. (2) more than 6,000 or less than 500 genes expressed. 32,626 cells were retained for further analysis after normalization. Harmony R package was applied for batch integration [19, 20]. Then cells were clustered into 15 cell populations by FindClusters function (dims = 1:18, resolution = 0.4). The tSNE and UMAP analysis were performed for reduction and visualization of gene expression. The signatures from previous publications and CellMarker [17, 21, 22] were used for annotation.

Tumor Immune Estimation Resource (TIMER2.0) (http://timer.cistrome.org/) [23] was used to analyze pan cancers expression of ACSL4. TIMER2.0 and CIBERSORT [24] were applied to search the relationship between ACSL4 transcriptional level and immune cell infiltration in CHOL.

Immunohistochemistry staining of ACSL4 was collected from The Human Protein Atlas (HPA) database (http://www.proteinatlas.org/) [25].

TISIDB (http://cis.hku.hk/TISIDB/) [26] was used to investigate the association of ACSL4 with immunostimulators, immunoinhibitors, chemokines (or receptors) in CHOL.

LinkedOmics (http://www.linkedomics.org/login.php) [27] was used to analyze co-expressed genes associated with transcription of the ACSL4 gene in CHOL.

### Human tissue samples

The tissue was from 40 CHOL patients who underwent surgery from 2013 to 2015 at The First Affiliated Hospital of Nanjing Medicine University. The patients were followed up until death or October 25, 2019. Our research was approved by the Ethics Committee of The Affiliated Hospital of Nanjing Medical University. The patients informed consent was obtained according to regional regulations.

### Real-time quantitative PCR (qPCR)

The total RNA of cells and tissues was extracted by RNA-Quick Purification Kit (Yishan, China). HiScript III 1st Strand cDNA Synthesis Kit (Vazyme, China) was used to reverse transcribe extracted RNA into cDNA. Then, qPCR was conducted with AceQ qPCR SYBR Green Master Mix (Vazyme, China) on the 7900HT Fast Real-Time PCR System (Applied Biosystems, MA, USA). The relative expression of ACSL4 was normalized to GAPDH by the 2^−ΔΔCt^ method. The primer sequences are listed as follows. ACSL4 forward: 5’-CATCCCTGGAGCAGATACTCT-3’, ACSL4 reverse: 5’-TCACTTAGGATTTCCCTGGTCC-3’; GAPDH forward: 5’-GGAGCGAGATCCCTC CAAAAT-3’, GAPDH reverse: 5’-GGCTGTTGTCATACTTCTCATGG-3’.

### Western blotting

RIPA Lysis Buffer (Beyotime, China) with PMSF is used to lyse tissue samples. Western blot was performed according to the manufacturer’s protocol. NcmECL Ultra (NCM Biotech, China) was applied to detect the level of protein. The following antibodies were used: anti-ACSL4 (22401-1-AP, Proteintech, China) and anti-GAPDH (ab8245, Abcam, UK).

### Cell culture and transfection

Human intrahepatic biliary epithelial cell line (HiBEC), human cholangiocarcinoma cell line RBE, HCCC9810, HuCCT1 and QBC939 were used in our study. All the cells were cultured in DMEM High Glucose (Biochannel, China) containing 10% fetal bovine serum (Gibco, USA) and 1% penicillin-streptomycin solution (Biochannel, China) at 37 °C with 5% CO2. Small interfering RNAs (siRNAs) were synthesized by GenePharma (Shanghai, China). Lipofectamine™ 2000 (11,668,019, Invitrogen, USA) was used for transfection following the manufacturer’s protocol. The following siRNA sequences were used: ACSL4 sense 5′-GAGGCUUCCUAUCUGAUUATT-3′ and anti-sense 5′-UAAUCAGAUAGGAAGCCUCTT-3′ [28].

### CCK8 assay

CCK8 assay kit (Dojindo, Japan) was used to assess the proliferation of cells. HuCCT1 or RBE cells transfected with negative control siRNA (si-NC) or si-ACSL4 were seeded in 96-well plates at 1 × 10^3^ cells/well the night before. On each day of the subsequent days, 100 µ medium containing 10 µ CCK8 reagent was added into each well. After 2 h incubation, the absorbance at 450 nm was measured by Multi-Mode Microplate Reader (Biotek, USA).

### EdU assay

EdU cell proliferation kit (Beyotime, China) was used for EdU assay to assess cell proliferation. HuCCT1 or RBE cells transfected with si-NC or si-ACSL4 were planted in 24-well plates at 80% confluence and cultured for 24 h, followed by 2 h incubation with 10µM EdU medium. The cells were fixed in 4% paraformaldehyde and permeabilized with 0.3% Triton in PBS for 15 min. Sequentially, the cells were stained with Alexa Fluor 555 azide for 30 min and DAPI for 10 min in the dark. The cells were photographed under a fluorescence microscope (Leica, Germany).

### Clone formation assay

HuCCT1 or RBE cells transfected with si-NC or si-ACSL4 were seeded in 6-well plates at 1 × 10^3^/well and incubated for 10 days. The cells were fixed and then stained with Crystal Violet Staining Solution (Beyotime, China) for 30 min. The colonies were counted and photographed.

### Transwell assay

Transwell assays were performed for migration. 5 × 10^4^ HuCCT1 or RBE cells transfected with si-NC or si-ACSL4 were seeded into Transwell BD Matrigel (Corning, USA). Serum-free medium was added to the upper layer and medium containing 20% fetal bovine serum was added to the lower layer. After 2 days incubation, migrating cells were fixed with 4% paraformaldehyde and then stained with Crystal Violet Staining Solution (Beyotime, China). The images were shot under a light microscope (Olympus, Japan).

### Wound healing assay

HuCCT1 or RBE cells transfected with si-NC or si-ACSL4 were seeded in 6-well plates at 5 × 10^5^ cells/well. When the density of cells reached 90-100%, we used a 200 µl pipette tip to scratch a straight wound. The images were taken by light microscopy (Olympus, Japan).

### ROS and iron assay

ROS level was measured by Reactive Oxygen Species Assay Kit (Biosharp, China). Intracellular ferrous iron (Fe^2+^) level was measured by FerroOrange assay kit (Dojindo, Japan). The fluorescence intensity was detected by Multi-Mode Microplate Reader (Biotek, USA).

### Statistical analysis

Data were analyzed using R 4.2.1 and GraphPad Prism 8. T-test was used for differences between the two groups and χ2 test was used to analyze correlations between ACSL4 expression and clinicopathological variables. Kaplan-Meier methods and log-rank test were applied to assess survival outcomes. Associations were evaluated using Spearman’s correlation analysis. Differences were considered statistically significant when *P < 0.05, **P < 0.01, or ***P < 0.001.

## Results

### The expression of ACSL4 is upregulated in CHOL

**Fig. 1:**
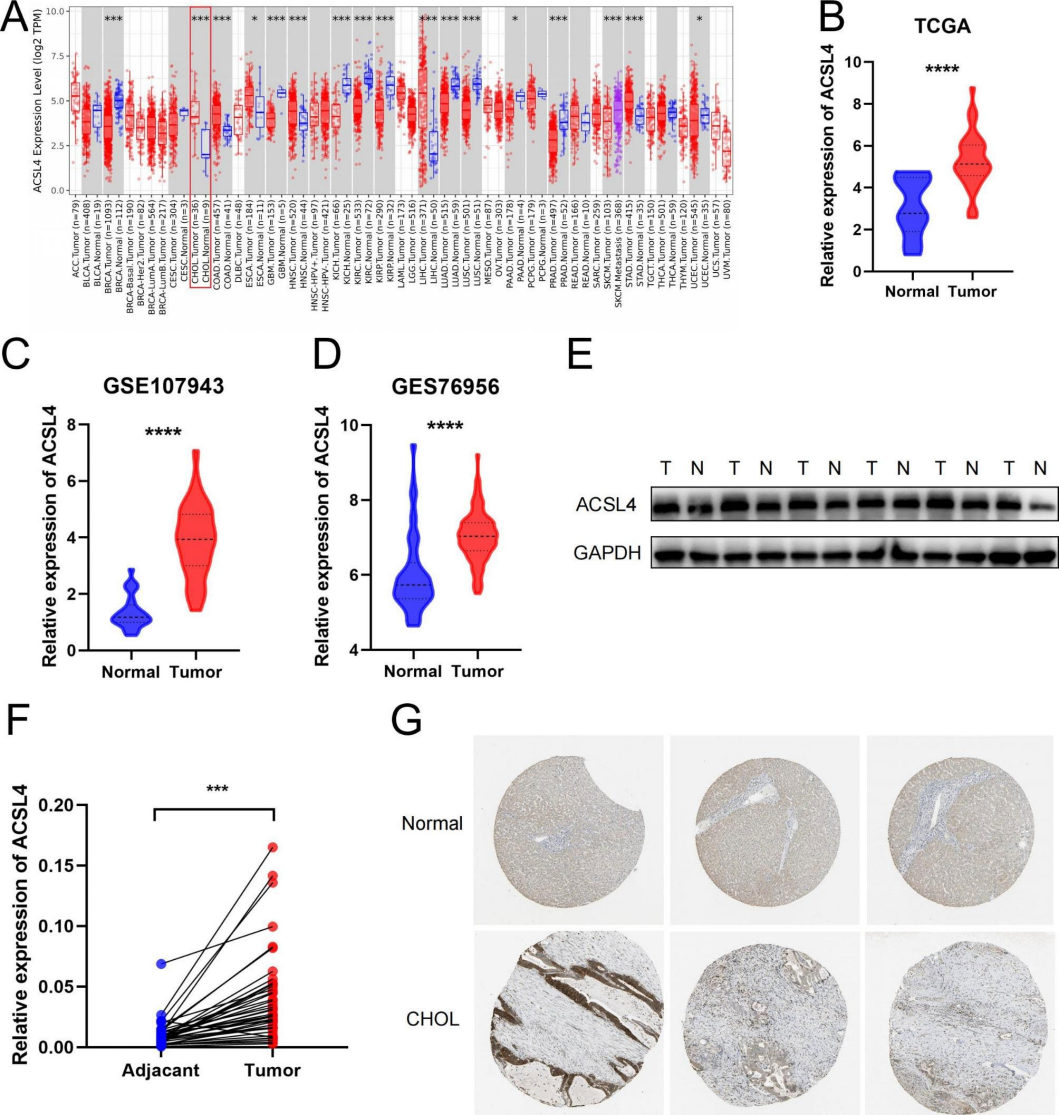
The expression of ACSL4 is upregulated in CHOL. **(A)** The expression of ACSL4 in pan cancers from TCGA database was analyzed by TIMER2.0. **(B-D)** The level of ACSL4 is upregulated in CHOL tissues compared with normal tissues in TCGA-CHOL, GSE107943, GSE76956 datasets. **(E)** Western blotting was used to detect the protein level of ACSL4 in 6 pairs of CHOL tumor tissues (T) and their paired normal tissues (N). **(F)** The mRNA level of ACSL4 in 40 pairs of CHOL tissues and their paired normal tissues. **(G)** The expression of ACSL4 in CHOL tissue was measured by IHC staining from HPA database

We used TIMER2.0 to explore the different expression of ACSL4 between normal and tumor tissue in various cancers (Fig. 1A). The results showed that the level of ACSL4 is increased in cholangiocarcinoma (CHOL), colon cancer, head and neck cancer, liver cancer and stomach cancer. Also, we verified that ACSL4 expression levels were upregulated in CHOL with TCGA-CHOL, GSE76297 and GSE107943 databases (Fig. 1B-D). Western Blot and qPCR were used to examine the expression of ACSL4 was examined in our CHOL patient’s tissues and paired adjacent tissues, and we found that ACSL4 protein (Fig. 1E) and mRNA levels (Fig. 1F) were higher in CHOL tissues than in paired adjacent tissues. IHC staining of ACSL4 in CHOL tissues and normal liver tissue from HPA database also confirmed this result (Fig. 1G). The above data demonstrated that ACSL4 was increased in CHOL tissue.

### Increased ACSL4 expression associated with poor prognosis in patients with CHOL

**Fig. 2:**
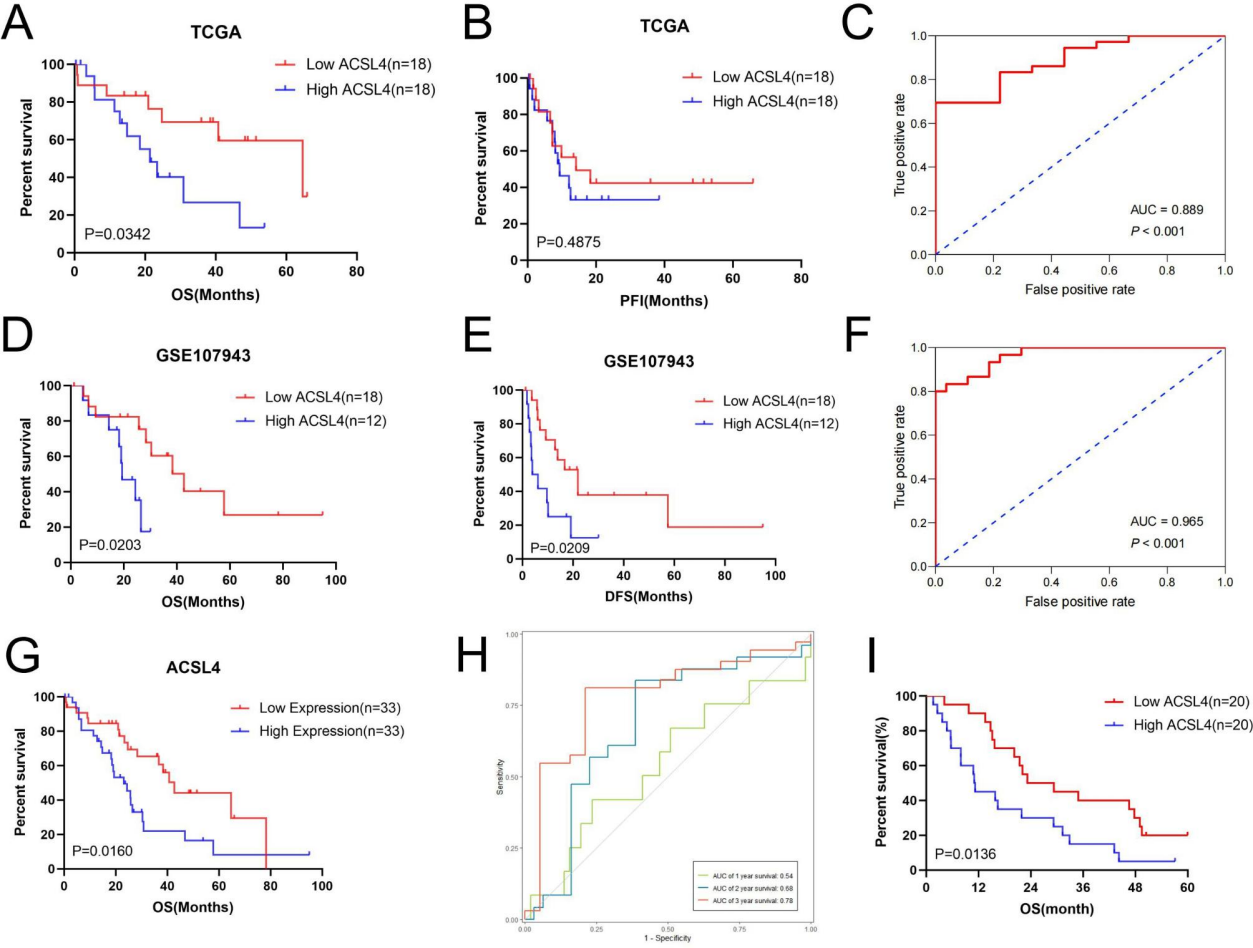
Increased ACSL4 mRNA expression associated with poor overall survival in patients with CHOL. **(A-B)** OS, PFI in high and low ACSL4 group in TCGA-CHOL cohort. **(C)** The ROC curve analysis of ACSL4 in TCGA-CHOL cohort. **(D-E)** OS, DFS in high and low ACSL4 group in GSE107943 cohort. **(F)** The ROC curve analysis of ACSL4 in GSE107943 cohort. **(G)** OS of ACSL4 expression in meta-dataset. **(H)** The time-dependent ROC curve analysis of ACSL4 in meta-dataset. **(I)** OS of ACSL4 expression in our cohort

To study the correlation between ACSL4 and the prognosis in CHOL, the patients in TCGA and GSE107943 datasets were divided into high ACSL4 group and low ACSL4 group separately, according to the level of ACSL4. Kaplan-Meier analysis showed that patients with high expression of ACSL4 exhibited poor overall survival (OS) (*P* = 0.0342, Fig. 2A) and progress free interval (PFI) (*P* = 0.4875, Fig. 2B) in TCGA database, but PFI was not statistically significant. The AUC of ROC analysis was used to measure the diagnostic value of the ACSL4 in CHOL (AUC = 0.889, *P* < 0.001, Fig. 2C). A similar result was found in GSE107943 (Fig. 2D-F). In addition, we combined TCGA-CHOL and GSE107943 dataset into a meta-dataset and then removed batch effects via the “sva” package [29]. In the new dataset, ACSL4 high expression group also means discouraging overall prognosis (*P* = 0.0160, Fig. 2G), and the AUC of time dependent ROC curve was 0.54, 0.68, and 0.78 for 1, 2, and 3 years, respectively (Fig. 2H). Also, in our 40 CHOL patients, high ACSL4 expression also means poor OS (*P* = 0.0136, Fig. 2I). Carbohydrate antigen 19 - 9 (CA19-9), which is the most widely used tumor marker in diagnosis of CHOL, is higher in the high ACSL4 expression group, though the *P*-value was not statistically significant (Table 1). Therefore, the level of ACSL4 can be used to predict long-term survival in CHOL patients.

**Table 1:** Relationship between ACSL4 and clinicopathological characteristics in CHOL patients

		ACSL4 expression			
Variable	High(N = 33)		Low(N = 33)		p value
Sex					0.6144
Male	19		21		
Female	14		12		
Age					0.4594
< 65	17		14		
≥ 65	16		19		
TNM stage					0.1593
I-II	22		27		
III-IV	11		6		
Status					0.0797
Death	23		16		
Alive	10		17		
CA19-9,IU/L	90.98 (1.2, 616.2)		667.4 (1.0, 6910.0)		0.0694

### Association between ACSL4 and immune infiltration in CHOL

**Fig. 3:**
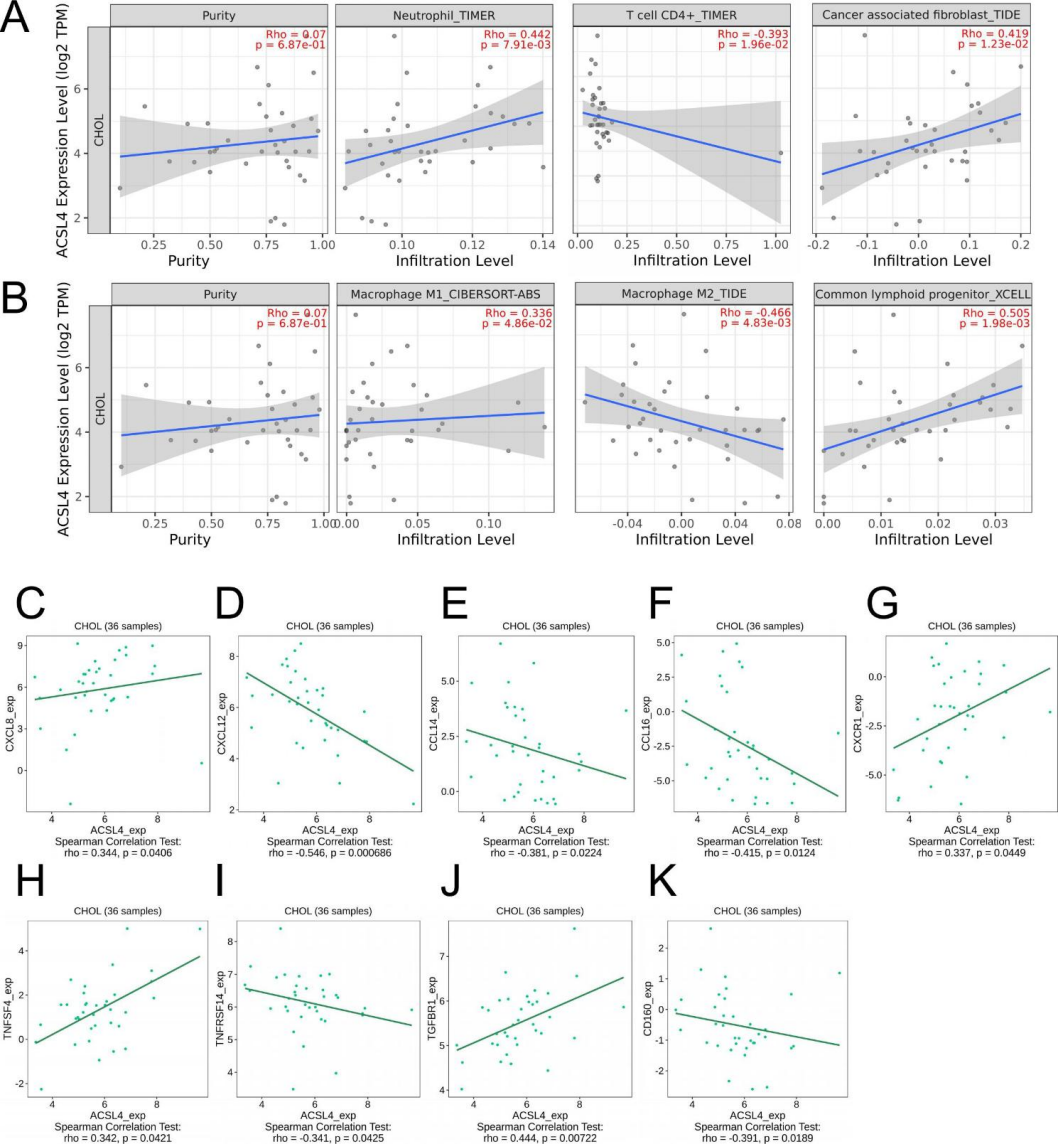
Association between ACSL4 and immune infiltration expression in CHOL. **(A-B)** Correlation of ACSL4 expression with infiltration levels of Neutrophil, CD4 + T cell, Cancer associated fibroblast cell, Macrophage M1, Macrophage M2, and Common lymphoid progenitor in CHOL available at TIMER2.0 database (N = 36). **(C-G)** Correlation between ACSL4 expression and chemokines and chemokines receptors in CHOL available at TISIDB database. **(H-K)** Correlation between ACSL4 expression and immunostimulatory and immunoinhibitory in CHOL available at TISIDB database

In recent years, many researches demonstrate that immune infiltration plays a key role of tumor progression. Therefore, TIMER2.0 was used to assess the association between ACSL4 expression and immune cell infiltration in CHOL. The results showed that ACSL4 was correlated with infiltrating degree of Neutrophil (Rho = 0.442, *P* = 0.00791), CD4 + T cell (Rho = -0.393, *P* = 0.0196), Cancer associated fibroblast cell (Rho = 0.419, *P* = 0.0123), Macrophage M1 (Rho = 0.336, *P* = 0.0486), Macrophage M2 (Rho = -0.466, *P* = 0.00483), and Common lymphoid progenitor (Rho = 0.505, *P* = 0.00198) (Fig. 3A-B). Then, we investigated the correlation between ACSL4 expression and various immune signatures in TISIDB database. For example, we found that ACSL4 expression was significantly linked with chemokines and chemokines receptors in CHOL, including CXCL8 (Rho = 0.344), CXCL12 (Rho = -0.546), CXCL14 (Rho = -0.381), CXCL16 (Rho = -0.415) , CXCR1 (Rho = 0.337) (Fig. 3C-G). Meanwhile, we found that ACSL4 expression was also correlated with immunostimulator and immunoinhibitor in CHOL, including TNFS4 (Rho = 0.342), TNFRSF14 (Rho = -0.341), TGFBR1 (Rho = 0.444), CD160 (Rho = -0.391) (Fig. 3H-K). Therefore, it was confirmed that ACSL4 may function as an immunoregulatory factor to regulate immune infiltration in CHOL.

### Relationship between ACSL4 expression and TILs in CHOL

**Fig. 4:**
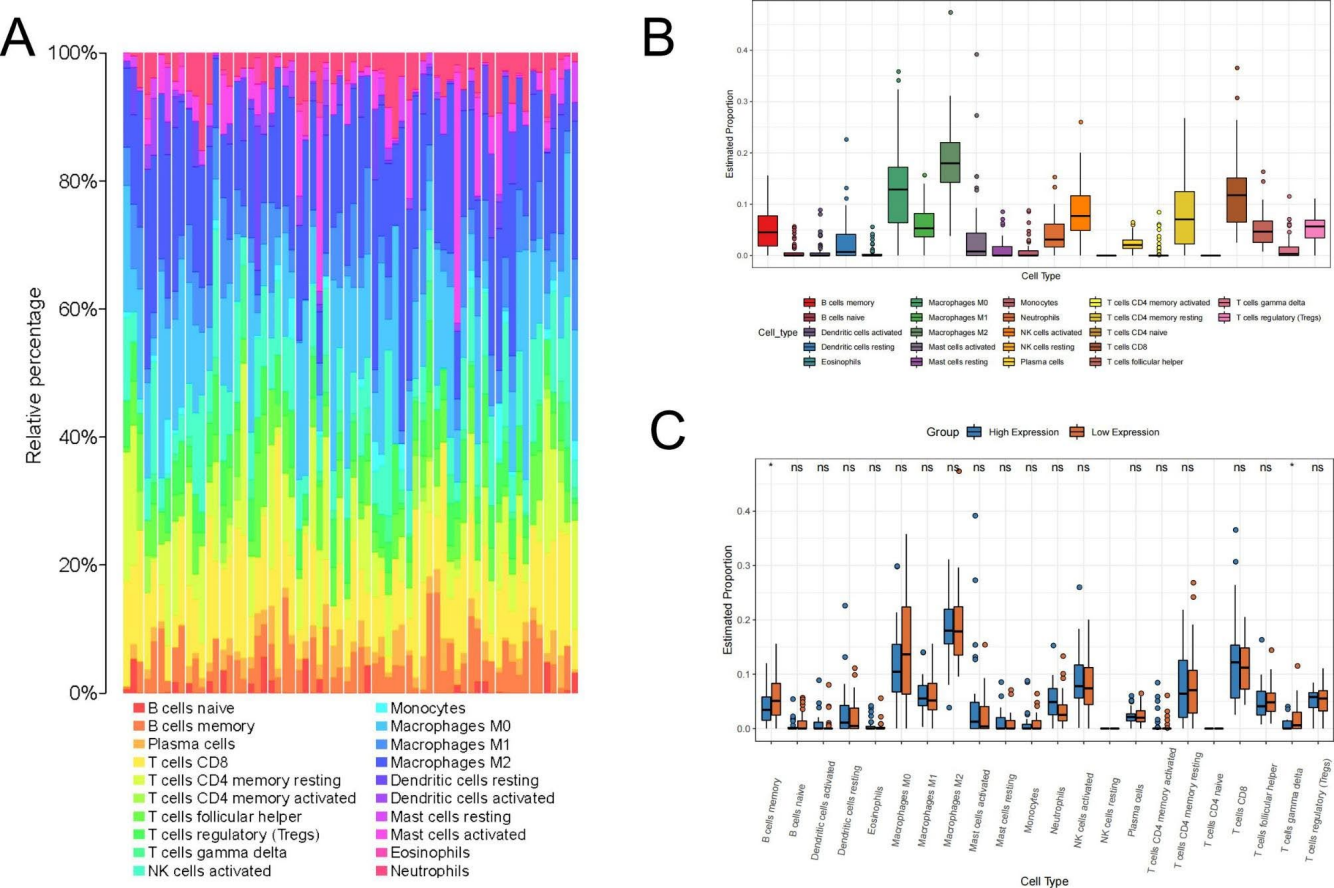
Relationship between ACSL4 expression and TILs in CHOL. **(A-B)** Relative proportions of 22 subtypes of tumor-infiltrating immune cells for each sample in CHOL by CIBERSORT. **(C)** Comparison of the immune cell fraction difference between the low and high ACSL4 expression groups

Tumor-infiltrating lymphocytes (TILs) are independent factors in the development of various cancers, including CHOL. CIBERSORT was employed to investigate the proportions of 22 immune cells in CHOL. we found that the level of M0 macrophages, M2 macrophages, active NK cells, resting CD4 + memory T cells and CD8 + T cells is increasing in CHOL (Fig. 4A-B). Then, the samples from TCGA and GSE107943 were divided into high and low ACSL4 expression group. The proportions of Neutrophils and CD8 + cell is upregulated in high ACSL4 expression groups. On the contrary, memory B cells, M0 macrophages and gamma delta (γδ) T cells are upregulated in the low ACSL4 expression group (Fig. 4C).

### ACSL4 expression and cells in CHOL were examined by single-cell analysis

**Fig. 5:**
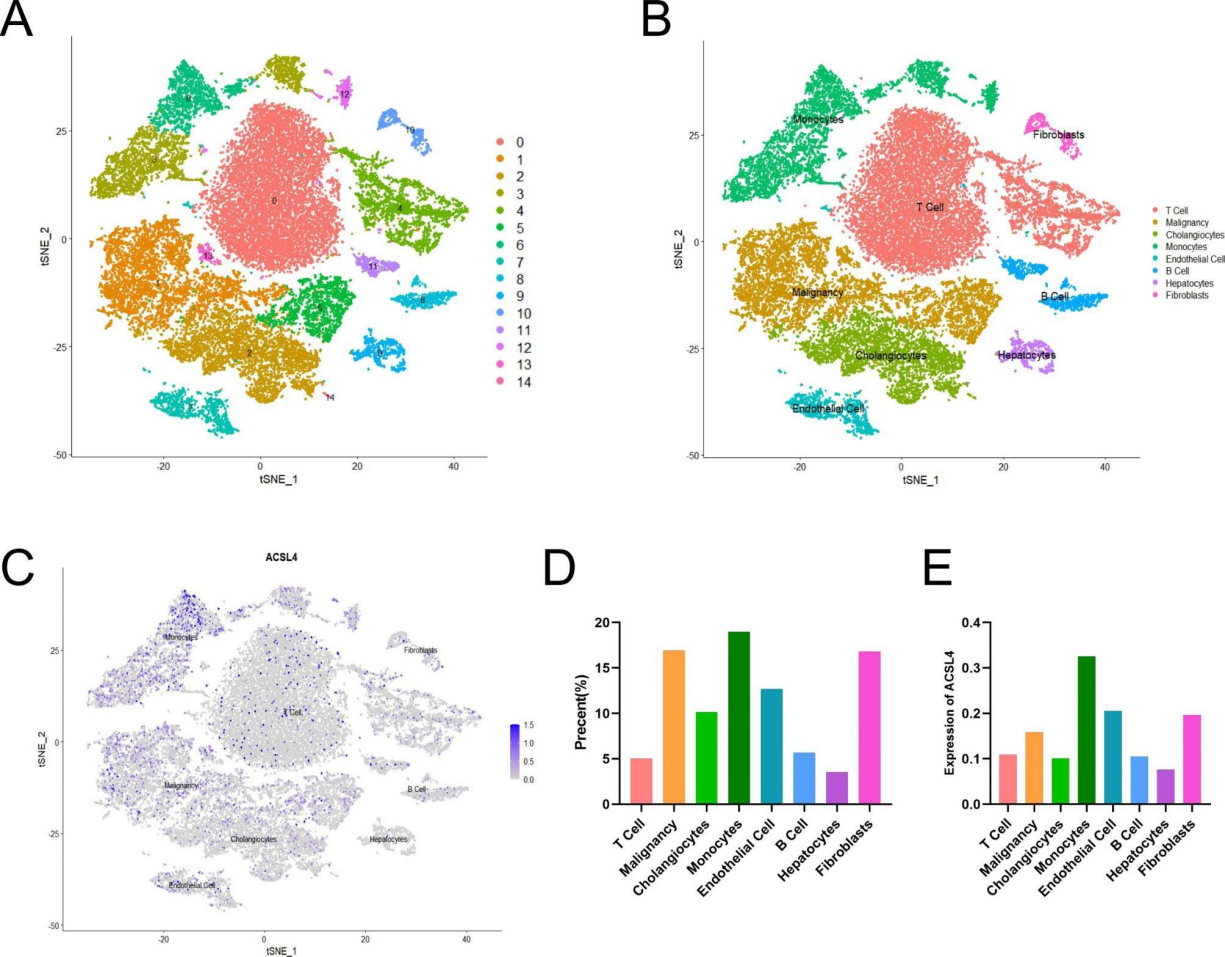
ACSL4 expression and cells in CHOL were examined by single-cell analysis. **(A)** Cell clusters for GSE138709 of five CHOL patients. **(B)** Cell markers for clusters’ annotation. **(C-E)** The ACSL4 expression in tissues extracted from the five CHOL patients

We analyzed the GSE138709 to examine the association between ACSL4 expression and cells. Cell populations were clustered into 15 groups, and 15 clusters were merged into 8 cell populations based on marker gene expression, which were T cells (CD3D), malignancy (KRT19), hepatocytes (ASGR1), cholangiocytes (FXYD2), monocytes (CD68), endothelial cells (FCN3), fibroblasts (ACTA2), and B cells (CD79A) (Fig. 5A-B, Supplement Fig. 2A-I). The results showed that ACSL4 could express in all 8 types of cells, especially in monocytes (Fig. 5C-E). And the expression of ACSL4 is upregulated mainly in malignancy, T cells and cholangiocytes in CHOL samples compared with normal samples (Supplement Fig. 2J).

### Co-expressed networks and potential function of ACSL4 in CHOL

**Fig. 6:**
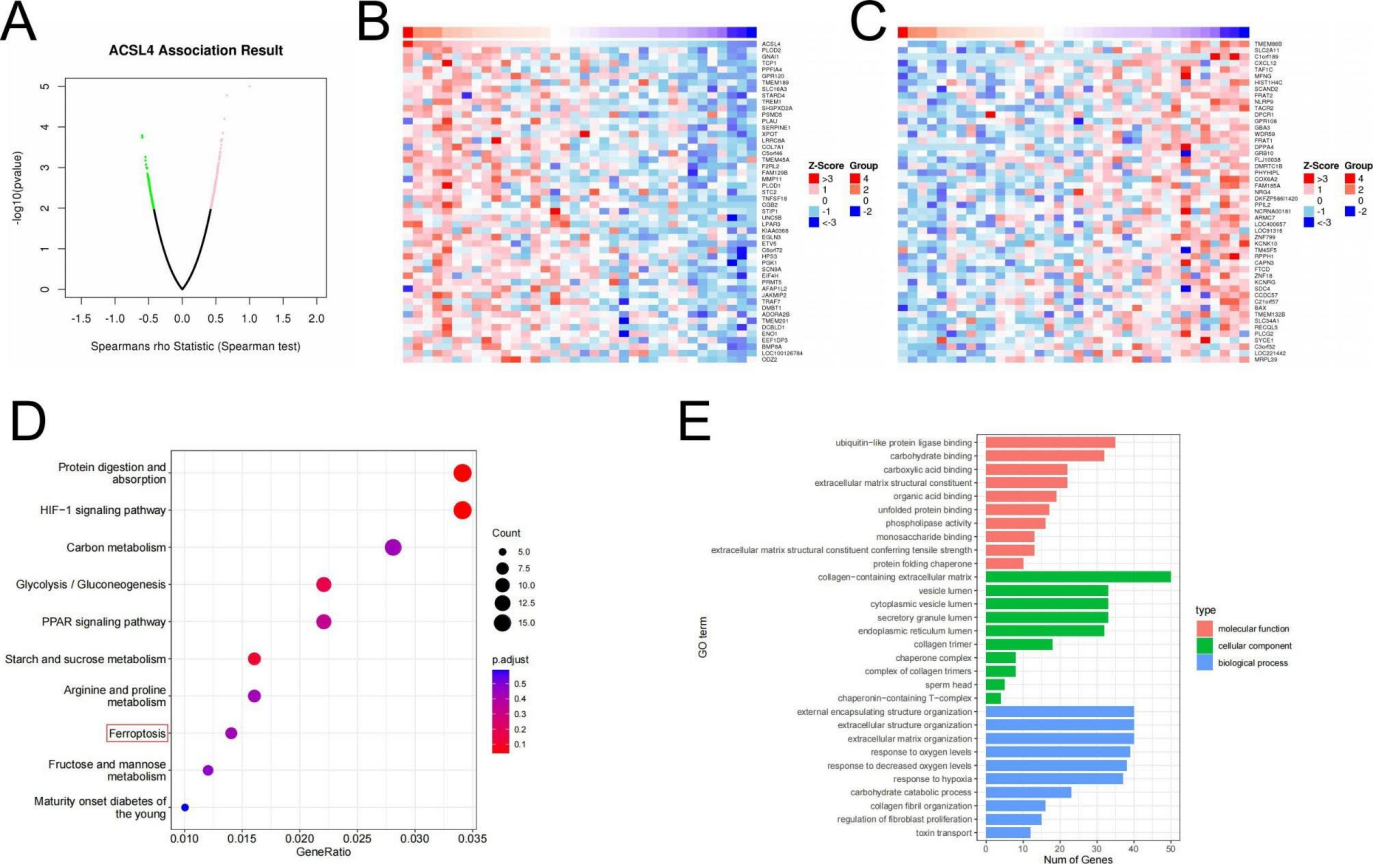
Co-Expressed Networks and Potential Function of ACSL4 in CHOL. **(A)** All genes co-expressed with ACSL4 in CHOL. **(B-C)** The top 50 positively correlated genes and the top 50 negatively correlated genes co-expressed with ACSL4 in CHOL. **(D)** KEGG pathway analysis of ACSL4 correlated genes in CHOL. **(E)** GO analysis of ACSL4 correlated genes in CHOL

To investigate the potential function and mechanism of ACSL4 in CHOL, Linkedomics was applied to analyze the co-expressed networks of ACSL4 in CHOL. We found that 192 genes were positively associated with ACSL4 and 114 genes were negatively associated with ACSL4 (*P* < 0.01, Fig. 6A). The heatmap showed the top 50 genes positive and negative correlation with ACSL4 in CHOL (Fig. 6B-C). Next, Kyoto Encyclopedia of Genes and Genomes (KEGG) pathway [30] and Gene Ontology (GO) analysis were analyzed in ACSL4 co-expressed genes (*P* < 0.05). The results showed that ACSL4 co-expressed genes were enriched in Protein digestion and absorption, HIF-1 signaling pathway and Carbon metabolism. Just as we expect, Ferroptosis was associated with ACSL4 co-expressed genes and we further certified that ACSL4 participated in the progress of ferroptosis in CHOL (Fig. 6D, Supplement Fig. 3A-F). ACSL4 co-expressed genes may involve in ubiquitin-like protein ligase binding, collagen-containing extracellular matrix, external encapsulating structure organization and other processes in GO term annotation (Fig. 6E). These results demonstrated that co-expressed genes of ACSL4 may mainly involve in metabolism-related pathway in CHOL.

### Knockdown of ACSL4 can significantly suppress the proliferation and migration in CHOL

**Fig. 7:**
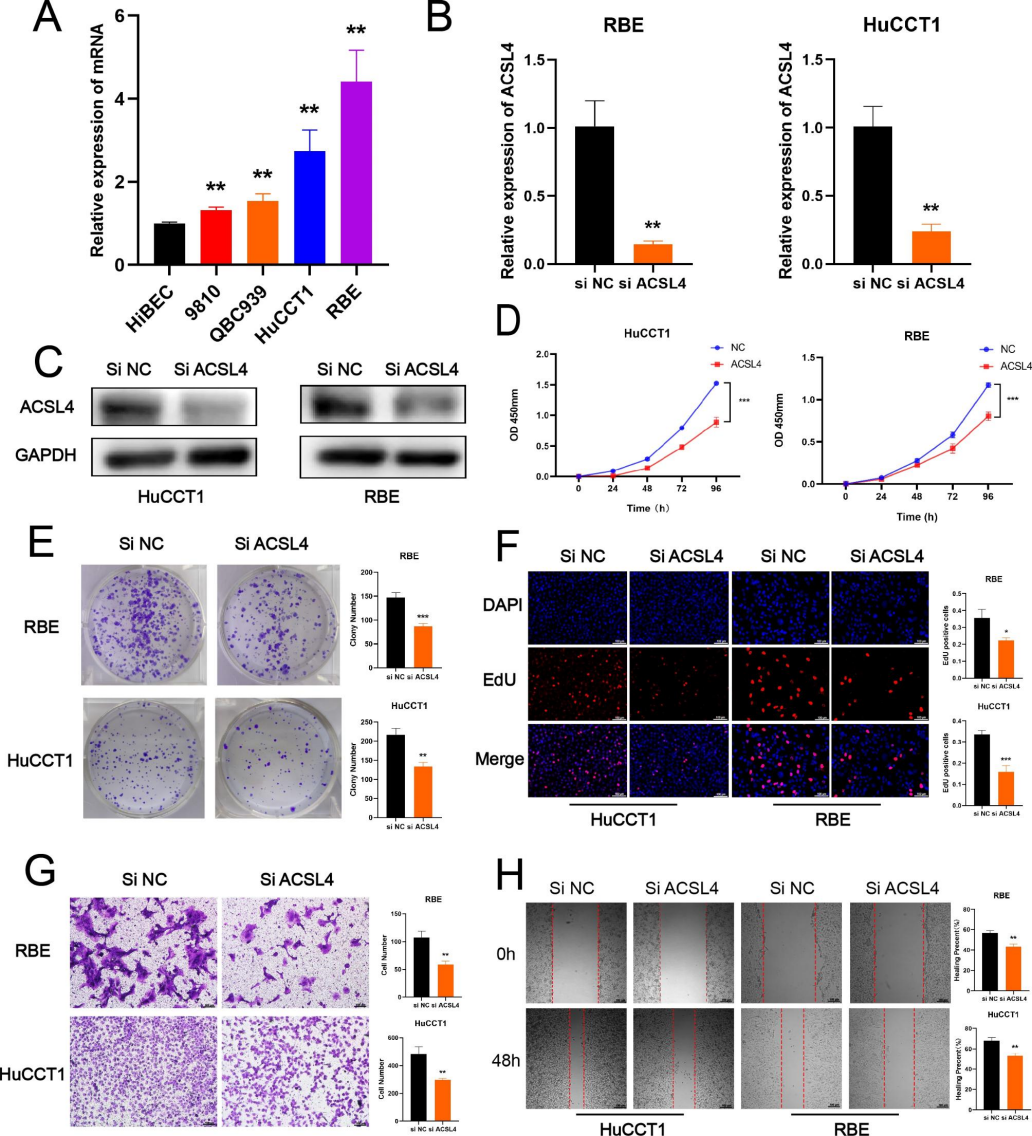
Knockdown of ACSL4 can significantly suppress the proliferation and migration in CHOL. **(A)** The mRNA expression of ACSL4 in BEC and 4 CHOL cell lines (9810, QBC939, HuCCT1 and RBE) was measured by qPCR. **(B-C)** The efficacy of ACSL4-siRNA was certified by qPCR and Western Blot in RBE and HuCCT1 cells. **(D-F)** The proliferation capacity was measured by CCK8 assay, clone formation assay and EdU assay in RBE and HuCCT1 cells with ACSL4 knockdown. **(G-H)** The ability of migration was measured by transwell assay and wound healing in RBE and HuCCT1 cells with ACSL4 knockdown. The *P* values were calculated using unpaired Student’s *t* test. Data were shown as mean ± SD. *P < 0.05; **P < 0.01; ***P < 0.001. NC, negative control. DAPI, 4’,6-diamidino-2-phenylindole. Scale bar: 100 μm

Based on the clinical evidence that ACSL4 has a potential role in CHOL tumor progression, we analyzed the expression of ACSL4 in BEC and 4 CHOL cell lines (Fig. 7A). We next selected RBE and HuCCT1 cells for siRNA-mediated knockdown experiments because expression was highest upregulated. The efficacy of ACSL4-siRNA was certified by qPCR and Western Blot (Fig. 7B-C). Then, cell viability was measured with CCK8, EdU and clone formation assays. The results indicated that knockdown of ACSL4 suppressed the proliferation of CHOL (Fig. 7D-F), and consistently, decreased the ability of migration by transwell and wound healing assay (Fig. 7G-H). These results indicated that ACSL4 knockdown can repress the proliferation and migration in cholangiocarcinoma.

## Discussion

In our previous study, we constructed a ferroptosis-related gene signature which could predict the prognosis of CHOL [12]. Five ferroptosis-related genes were selected (ACSL3, ACSL4, MUC1, SLC38A1 and SLC7A11) by univariate Cox regression and Lasso regression. We investigated the association between the expression of five genes and prognosis in our meta dataset. We found high ACSL4 or MUC1 expression in patients indicated poor overall survival, while the other 3 genes have no significant prognosis value (Supplement Fig. 1C-F). ACSL4, is one of long-chain acyl-coenzyme A synthases (ACSLs) family members, can behave as a crucial component for lipid metabolism and ferroptosis. As previous reported, ACSL4 could promote or suppress tumor growth depending on the specific types of cancer. In gastric cancer [31], glioma [32], lung adenocarcinoma [33] and breast cancer [34], ACSL4 is regarded as a tumor-suppressive factor. However, ACSL4 also can promote cancer progression in HCC [7] and colon cancer [8]. But, the role of ACSL4 in development of CHOL is still unclear. Therefore, we aimed to explore the functions of ACSL4 in the process of CHOL development.

At the beginning of this study, we analyzed the expression of ACSL4 in pan-cancer and the results showed that ACSL4 was differential expression gene in most cancers, including CHOL. The expression of ACSL4 is upregulated in CHOL tissues compared with normal tissues in the RNA-seq from TCGA and GEO datasets. At the same time, in our clinical samples, we observed the mRNA and protein level of ACSL4 is higher in CHOL samples than in paired adjacent samples by qPCR and Western Blot. Based on HPA database, immunohistochemical analysis showed strong ACSL4 staining in tumor tissues. Thus, we confirmed that ACSL4 expression is upregulated in CHOL tumor tissues.

In TCGA, GSE107943 and meta dataset, we found that elevated ACSL4 expression was consistent with poor prognosis in patients with CHOL. ROC curve analysis confirmed that ACSL4 had clinical diagnostic values for CHOL patients.

Recently, more and more studies reported that tumor immune infiltration cells had interactions with cancer progression [35, 36]. The expression of ACSL4 is linked to the level of CD8 + T cell infiltration and affects subsequent anti-tumor immunity [37, 38]. Whereas, whether ACSL4 is related to immune infiltration in CHOL remains completely unclear. Therefore, we analyzed the correlation between ACSL4 expression and immune infiltration in CHOL by TIMER2.0, TISIDB and CIBERSORT. This study discovered that ACSL4 is possible to regulate immune infiltration in CHOL. ACSL4 expression had a correlation with immune cells including CD4 + T cells, neutrophils, cancer associated fibroblasts (CAFs), macrophages and common lymphoid progenitors. Previous studies proved that tumor associated macrophages (TAMs), neutrophils and cancer associated fibroblasts are the important immune cells contributing to tumoral progression in CHOL [39–41]. Besides, ACSL4 expression was related to some immunostimulators, immunoinhibitors, chemokines, and receptors. Then, we explored the 22 types of TILs in CHOL and divided CHOL patients into high and low ACSL4 expression groups. Then we detected that a low level of B cell memory and γδ T cells was present in the high ACSL4 expression group in CHOL. These results indicated ACSL4 may regulate immune microenvironment through immune cells or TILs in CHOL. But these results need specific experiments to be further verified.

Furthermore, we analyzed the scRNA-seq profiles from GSE138709 to investigate the heterogeneity of ACSL4 expression in different cells in CHOL and normal samples. We divided 15 cell clusters into 8 types of cells, including T cells, malignancy, cholangiocytes, monocytes, hepatocytes, endothelial cells, fibroblasts, and B cells. The results showed that ACSL4 could express in all types of cells. And the upregulation of ACSL4 is mainly in malignancy, T cells and cholangiocytes in CHOL samples. These finding indicated that the mechanism of ACSL4 interaction with these cells need to be further explored to explain the potential functions of ACSL4 in CHOL development.

Moreover, we studied the co-expressed genes with ACSL4 in CHOL by Linkedomics to investigate the potential function of ACSL4 in CHOL. These ACSL4 co-expressed genes were enriched by KEGG pathways and GO terms, the results showed that these genes were mainly involved in Protein digestion and absorption, HIF-1 signaling pathway, Carbon metabolism, Glycolysis / Gluconeogenesis, PPAR signaling pathway, Starch and sucrose metabolism, Arginine and proline metabolism and Ferroptosis. These findings indicated that ACSL4 and its co-expressed genes were possible to play a crucial role in the process of metabolism in CHOL, just like in HCC [42]. ACSL4 is an enzyme that plays a role in lipid metabolism, which ferroptosis critically depends on [4, 5].Based on our previous study [12], we want to confirm whether ACSL4 is also a key factor of ferroptosis in CHOL. So, we used erastin (a ferroptosis activator) to provoke ferroptosis in RBE or HuCCT1, and the results showed that ACSL4 knockdown could reverse cell death and accumulation of ROS induced by erastin, but the level of Fe^2+^ did not significantly change (Supplement Fig. 3). From these results, we think that ACSL4 is crucial for ferroptosis in CHOL. But ACSL4-dependent ferroptosis does not account for the tumor promotion of ACSL4 in CHOL, just like the previous study in HCC [43]. Interestingly, ferroptosis has a dual role in promoting and suppressing tumor in tumorigenesis. Ferroptosis can promote cancer by triggering immune response for ferroptotic damage within the tumor microenvironment [44, 45]. Meanwhile, we can’t exclude the impact of other ACSL4-dependent biological process in CHOL development. Besides, the excessive accumulation of lipid peroxides is recognized as a decisive factor of ferroptosis [3, 13]. ACSL4 can promote the progression of HCC via reprograms fatty acid metabolism [42]. Therefore, we hypothesize that ACSL4 can also promote the process of CHOL by regulating lipid accumulation and metabolism, but it needs more experiments to verify.

In vitro, the expression of ACSL4 is also higher in 4 CHOL cell lines than that of BEC. To further validate the function of ACSL4 in CHOL, through siRNA was transfected to knockdown of ACSL4 in 2 CHOL cell lines (RBE and HuCCT1). The results indicated that knockdown of ACSL4 could suppress the ability of proliferation and migration in CHOL. Therefore, ACSL4 is a crucial tumorigenic factor in CHOL development in vitro.

In conclusion, this study is the first research exploring the function of ACSL4 in CHOL. The level of ACSL4 was elevated in CHOL. High expression of ACSL4 was consistent with poor prognosis and ACSL4 had significant diagnostical value in CHOL. Furthermore, we found that ACSL4 is associated with immune infiltration and TILs in CHOL, and we explored the expression of ACSL4 in CHOL by single-cell analysis. Additionally, we investigated potential function of ACSL4 and its co-expressed genes in CHOL. In vitro, we verified that ACSL4 could promote the development of CHOL by knockdown of ACSL4. These findings indicated that ACSL4 may be a potential target for anti-cancer strategies in CHOL, and additional researches were encouraged to identify the specific mechanism of ACSL4 in regulate the development of CHOL.

## Electronic supplementary material

Below is the link to the electronic supplementary material.

Supplementary Material 1

## Data Availability

The original contributions presented during this study are included in this published article. The datasets analyzed during the current study are available in the GEO repository (https://www.ncbi.nlm.nih.gov/geo, GEO accession number: GSE76297, GSE107943 and GSE138709), TCGA repository (https://www.cancer.gov/tcga), TIMER2.0 repository (http://timer.cistrome.org/), HPA repository (http://www.proteinatlas.org/), TISIDB repository (http://cis.hku.hk/TISIDB/), LinkedOmics repository (http://www.linkedomics.org/login.php).

## Abbreviations

ACSL3: Acyl-CoA synthetase long-chain family member 3
ACSL4: Acyl-CoA synthetase long-chain family member 4
CA19-9: Carbohydrate antigen 19 − 9
CAFs: Cancer associated fibroblasts
CHOL: Cholangiocarcinoma
DAPI: 4’,6-diamidino-2-phenylindole
GEO: Gene Expression Omnibus
GO: Gene Ontology
HCC: Hepatocellular carcinoma
KEGG: Kyoto Encyclopedia of Genes Genomes
MUC1: Mucin 1
qPCR: Real-time quantitative PCR
SLC38A1: Solute Carrier Family 38 Member 1
SLC7A11: Solute Carrier Family 7 Member 11
TCGA: The Cancer Genome Atlas
TAMs: Tumor associated macrophages
TILs: Tumor-infiltrating lymphocytes

## References

[1] Brindley PJ (2021). Cholangiocarcinoma Nat Rev Dis Primers.

[2] Banales JM (2020). Cholangiocarcinoma 2020: the next horizon in mechanisms and management. Nat Rev Gastroenterol Hepatol.

[3] Jiang X, Stockwell BR, Conrad M (2021). Ferroptosis: mechanisms, biology and role in disease. Nat Rev Mol Cell Biol.

[4] Doll S (2017). ACSL4 dictates ferroptosis sensitivity by shaping cellular lipid composition. Nat Chem Biol.

[5] Gan B (2022). ACSL4, PUFA, and ferroptosis: new arsenal in anti-tumor immunity. Signal Transduct Target Ther.

[6] Ndiaye H et al. *Immunohistochemical staining reveals differential expression of ACSL3 and ACSL4 in hepatocellular carcinoma and hepatic gastrointestinal metastases*. Biosci Rep, 2020. 40(4). 10.1042/BSR20200219 PMC7198044 32286604

[7] Chen J (2020). ACSL4 promotes hepatocellular carcinoma progression via c-Myc stability mediated by ERK/FBW7/c-Myc axis. Oncogenesis.

[8] Sanchez-Martinez R (2017). Complementary ACSL isoforms contribute to a non-warburg advantageous energetic status characterizing invasive colon cancer cells. Sci Rep.

[9] Sirica AE, Gores GJ (2014). Desmoplastic stroma and cholangiocarcinoma: clinical implications and therapeutic targeting. Hepatology.

[10] Fabris L (2019). The tumour microenvironment and immune milieu of cholangiocarcinoma. Liver Int.

[11] Wu XG (2021). Identification and validation of the Signatures of infiltrating Immune cells in the Eutopic Endometrium Endometria of Women with Endometriosis. Front Immunol.

[12] Wang Z (2022). Identification of a ferroptosis-related gene signature for predicting the prognosis of cholangiocarcinoma. Expert Rev Gastroenterol Hepatol.

[13] Dixon SJ (2012). Ferroptosis: an iron-dependent form of nonapoptotic cell death. Cell.

[14] Park SY (2009). Expression of MUC1, MUC2, MUC5AC and MUC6 in cholangiocarcinoma: prognostic impact. Oncol Rep.

[15] Chaisaingmongkol J (2017). Common molecular subtypes among asian Hepatocellular Carcinoma and Cholangiocarcinoma. Cancer Cell.

[16] Ahn KS (2019). Prognostic subclass of intrahepatic cholangiocarcinoma by integrative molecular-clinical analysis and potential targeted approach. Hepatol Int.

[17] Zhang M (2020). Single-cell transcriptomic architecture and intercellular crosstalk of human intrahepatic cholangiocarcinoma. J Hepatol.

[18] Stuart T (2019). Comprehensive Integration of single-cell data. Cell.

[19] Korsunsky I (2019). Fast, sensitive and accurate integration of single-cell data with Harmony. Nat Methods.

[20] Tran HTN (2020). A benchmark of batch-effect correction methods for single-cell RNA sequencing data. Genome Biol.

[21] Chen Z (2021). PNOC expressed by B cells in Cholangiocarcinoma was Survival related and LAIR2 could be a T cell exhaustion biomarker in Tumor Microenvironment: characterization of Immune Microenvironment combining single-cell and bulk sequencing technology. Front Immunol.

[22] Zhang X (2019). CellMarker: a manually curated resource of cell markers in human and mouse. Nucleic Acids Res.

[23] Li T (2020). TIMER2.0 for analysis of tumor-infiltrating immune cells. Nucleic Acids Res.

[24] Newman AM (2015). Robust enumeration of cell subsets from tissue expression profiles. Nat Methods.

[25] Uhlen M et al. *A pathology atlas of the human cancer transcriptome*. Science, 2017. 357(6352). 10.1126/science.aan2507 28818916

[26] Ru B (2019). TISIDB: an integrated repository portal for tumor-immune system interactions. Bioinformatics.

[27] Vasaikar SV (2018). LinkedOmics: analyzing multi-omics data within and across 32 cancer types. Nucleic Acids Res.

[28] Li Y (2019). Ischemia-induced ACSL4 activation contributes to ferroptosis-mediated tissue injury in intestinal ischemia/reperfusion. Cell Death Differ.

[29] Leek JT (2012). The sva package for removing batch effects and other unwanted variation in high-throughput experiments. Bioinformatics.

[30] Kanehisa M (2023). KEGG for taxonomy-based analysis of pathways and genomes. Nucleic Acids Res.

[31] Ye X (2016). Tumor-suppressive functions of long-chain acyl-CoA synthetase 4 in gastric cancer. IUBMB Life.

[32] Cheng J (2020). ACSL4 suppresses glioma cells proliferation via activating ferroptosis. Oncol Rep.

[33] Zhang Y (2021). High-fat diet impairs ferroptosis and promotes cancer invasiveness via downregulating tumor suppressor ACSL4 in lung adenocarcinoma. Biol Direct.

[34] Kwon YS (2021). Acyl-CoA synthetase-4 mediates radioresistance of breast cancer cells by regulating FOXM1. Biochem Pharmacol.

[35] Garaud S et al. *Tumor infiltrating B-cells signal functional humoral immune responses in breast cancer*. JCI Insight, 2019. 5. 10.1172/jci.insight.129641 PMC6795287 31408436

[36] Li B (2016). Comprehensive analyses of tumor immunity: implications for cancer immunotherapy. Genome Biol.

[37] Liao P (2022). CD8(+) T cells and fatty acids orchestrate tumor ferroptosis and immunity via ACSL4. Cancer Cell.

[38] Luo W (2021). ACSL4 expression is Associated with CD8 + T cell infiltration and Immune response in bladder Cancer. Front Oncol.

[39] Sirica AE, Campbell DJ, Dumur CI (2011). Cancer-associated fibroblasts in intrahepatic cholangiocarcinoma. Curr Opin Gastroenterol.

[40] Mao ZY (2015). Prognostic value of neutrophil distribution in cholangiocarcinoma. World J Gastroenterol.

[41] Subimerb C (2010). Tissue invasive macrophage density is correlated with prognosis in cholangiocarcinoma. Mol Med Rep.

[42] Chen J (2021). ACSL4 reprograms fatty acid metabolism in hepatocellular carcinoma via c-Myc/SREBP1 pathway. Cancer Lett.

[43] Grube J (2022). ACSL4-dependent ferroptosis does not represent a tumor-suppressive mechanism but ACSL4 rather promotes liver cancer progression. Cell Death Dis.

[44] Chen X (2021). Broadening horizons: the role of ferroptosis in cancer. Nat Rev Clin Oncol.

[45] Dai E (2020). Autophagy-dependent ferroptosis drives tumor-associated macrophage polarization via release and uptake of oncogenic KRAS protein. Autophagy.

