# Supplementary material for: ACSL4 serves as a novel prognostic biomarker correlated with immune infiltration in Cholangiocarcinoma
Source: BMC Cancer. 2023 May 16;23:444. doi: 10.1186/s12885-023-10903-5 (PMC10186676; doi:10.1186/s12885-023-10903-5)

ACSL4 serves as a novel prognostic biomarker correlated with immune infiltration in Cholangiocarcinoma

Shuochen Liu[1,2,†], Shilong Fan[1,2,†], Yirui Wang[1,2,†], Ruixiang Chen[1,2,†], Ziyi Wang[1,2,3], Yaodong Zhang[1,2,] Wangjie Jiang[1,2], Yananlan Chen[1,2], Xiao Xu[1,2], Yue Yu[1,2,*], Changxian Li[1,2,*], Xiangcheng Li[1,2,*].

1 Hepatobiliary Center, The First Affiliated Hospital of Nanjing Medical University, Nanjing, China.
2 Key Laboratory of Liver Transplantation, Chinese Academy of Medical Sciences, Nanjing, China.
3 Department of Liver Surgery and Transplantation, Liver Cancer Institute and Zhongshan Hospital, Fudan University, Shanghai, China.
*Correspondence: Xiangcheng Li, drxcli@njmu.edu.cn ; Changxian Li, doclicx20@163.com; Yue Yu, yuyue@njmu.edu.cn.
† These authors contribute equally to this work.

Supplement Figure

Supplement Figure 1 The relationship between 4 ferroptosis-related genes and overall survival in CHOL.

(A) The PCA analysis of TCGA-CHOL and GSE107943 datasets before removing batch effects. **(B)** The PCA analysis of TCGA-CHOL and GSE107943 datasets after removing batch effects. **(C-F)** OS in high and low MUC1, SLC7A11, ACSL3, SLC38A1 group in meta cohort.

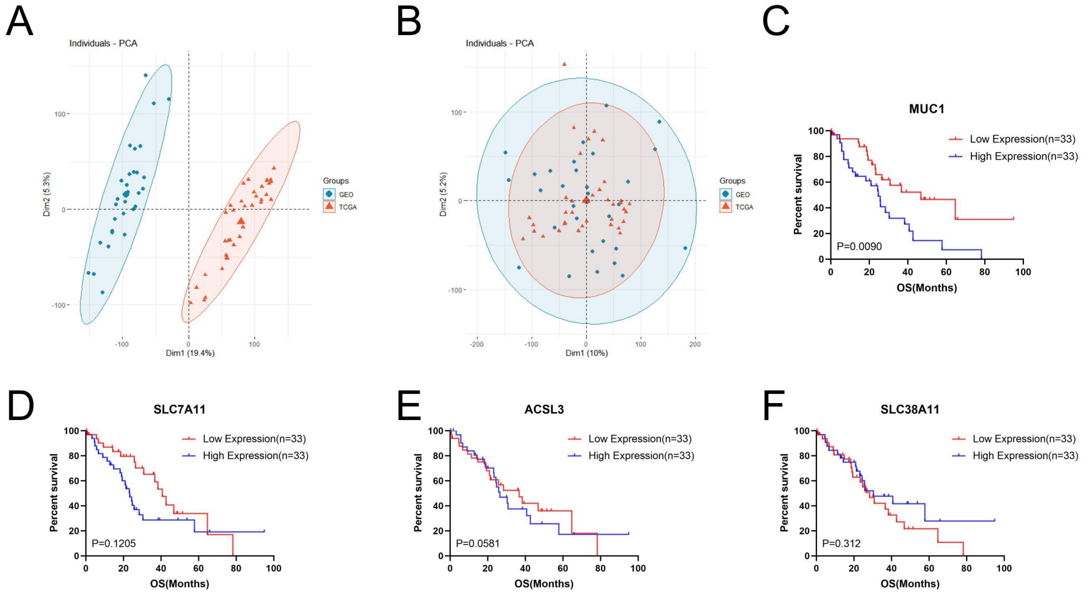

Supplement Figure 2 Cellular identity in GSE138709

(A-I) The expression of canonical marker genes for ten distinct cell types is shown in tSNE plots. T cells (CD3D), malignancy (KRT19), cholangiocytes (FXYD2), monocytes (CD14), hepatocytes (ASGR1), endothelial cells (FCN3), fibroblasts (ACTA2), and B cells (CD79A). **(J)** The expression of ACSL4 in different types of cells in CHOL compared with normal samples.

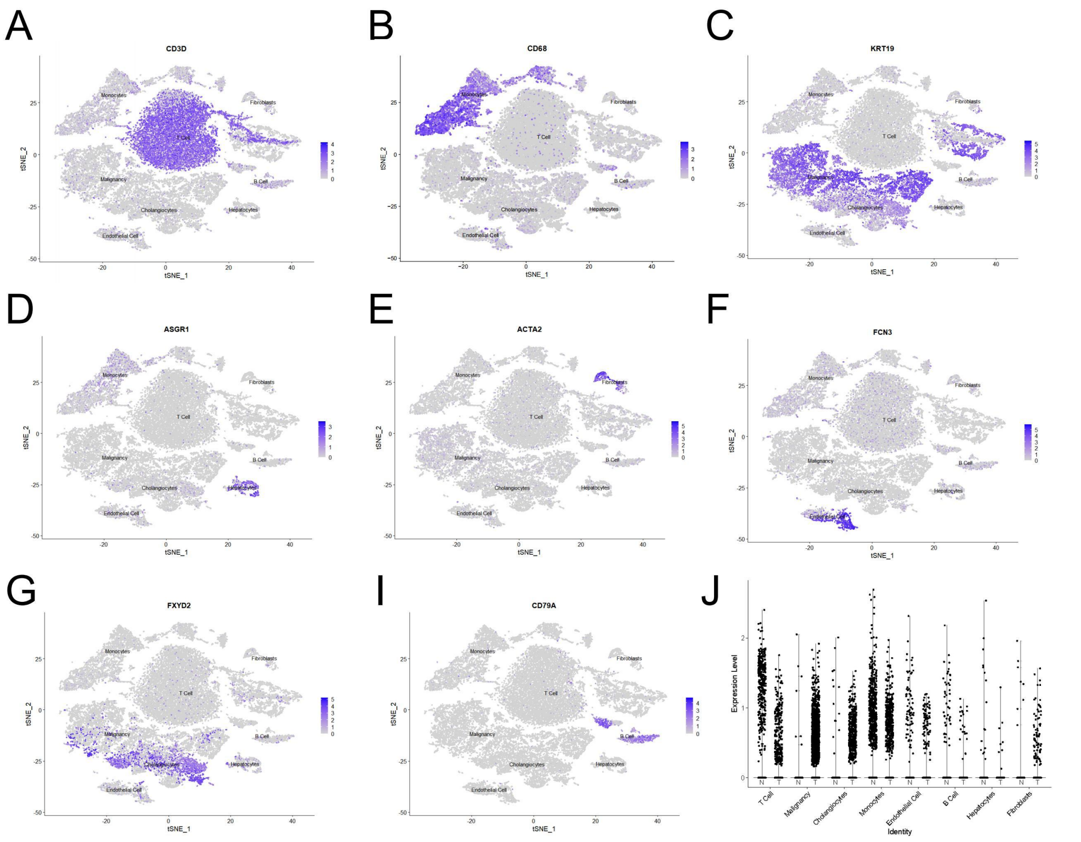

Supplement Figure 3 ACSL4 acts as a crucial factor of ferroptosis in CHOL

(A-B) Knockdown of ACSL4 alleviated erastin-induced cell death in RBE and HuCCT1. **(C-F)** ROS and Fe^{2+} were assayed in RBE and HuCCT1 following treatment with erastin (20 μM) for 24 h.

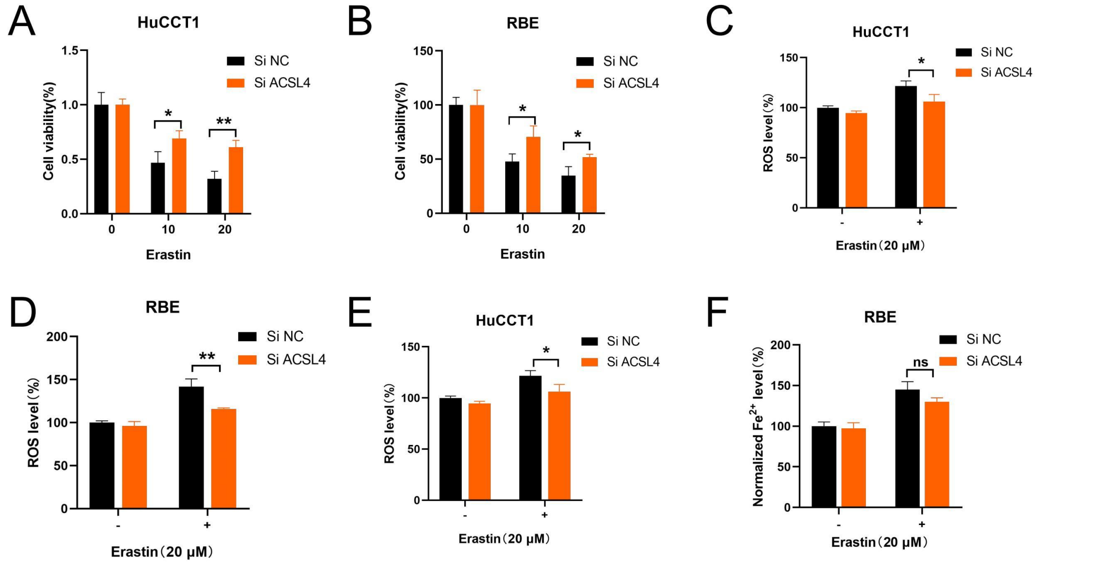

Figure 1E

ACSL4

T N T N T N T N T N T N

ACSL4 79kb

70kb

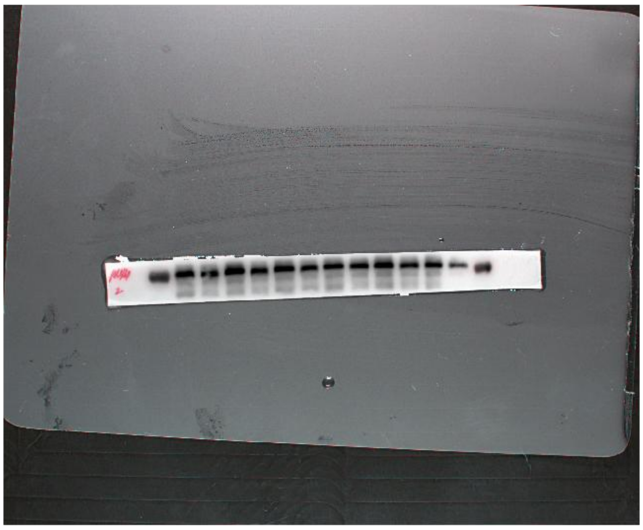

The other exposure image

T N T N T N T N T N T N

ACSL4 79kb

70kb

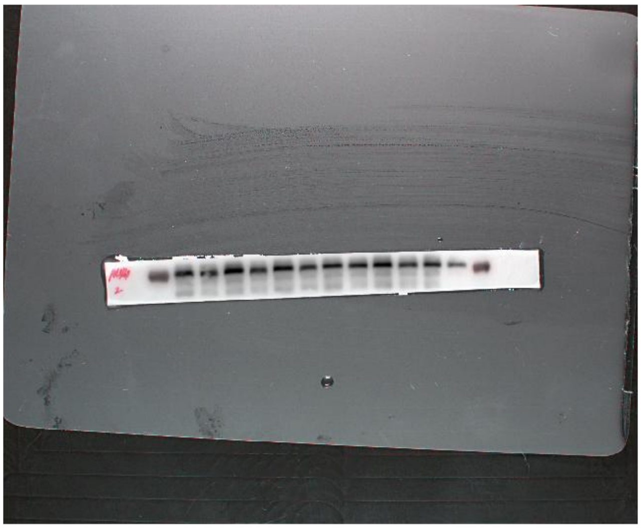

GAPDH

T N T N T N T N T N T N

GAPDH 36kb

40kb

35kb

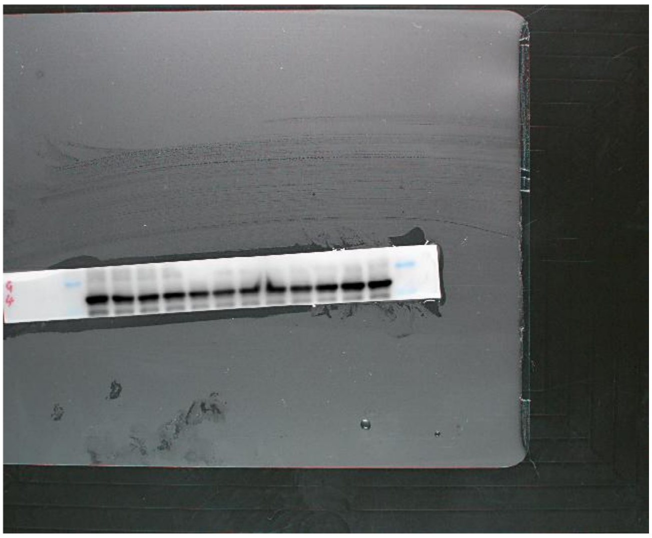

The other exposure image

T N T N T N T N T N T N

GAPDH 36kb

40kb

35kb

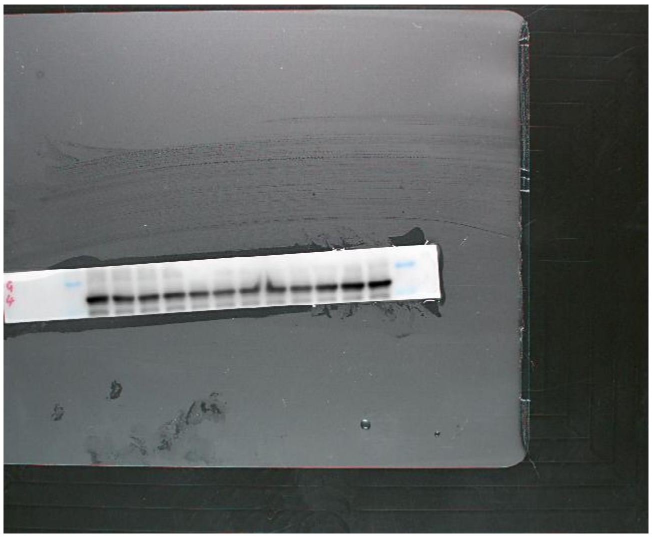

Figure 7E

HuCCT1

Si NC Si ACSL4 ACSL4 79kb

100kb
70kb

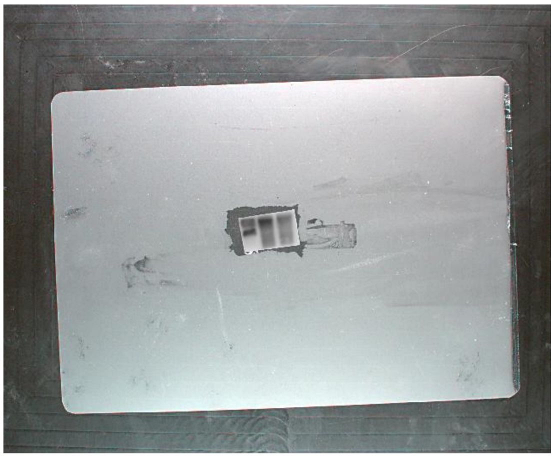

RBE

Si NC Si ACSL4 ACSL4 79kb

100kb
70kb

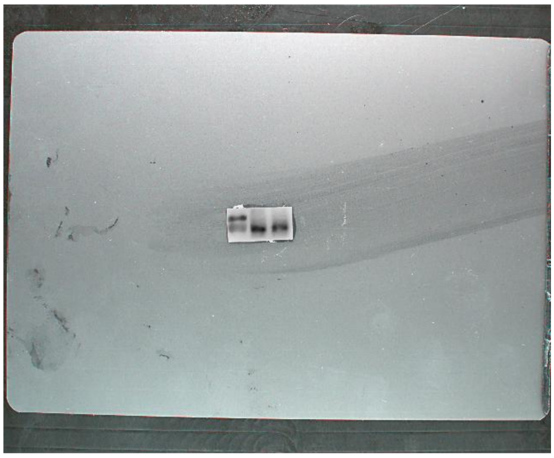

HuCCT1

Si NC Si ACSL4 GDPDH 36kb

40kb
35kb

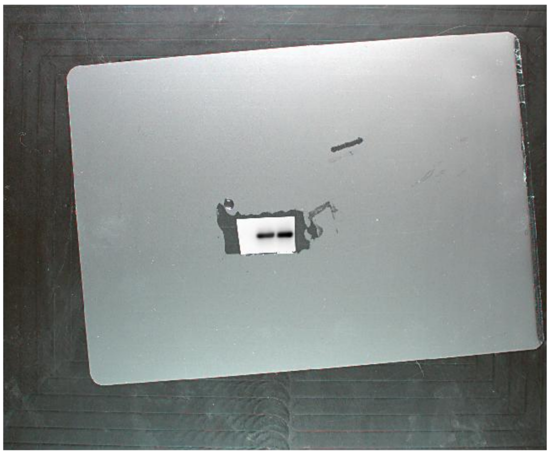

RBE

Si NC Si ACSL4 GDPDH 36kb

40kb
35kb

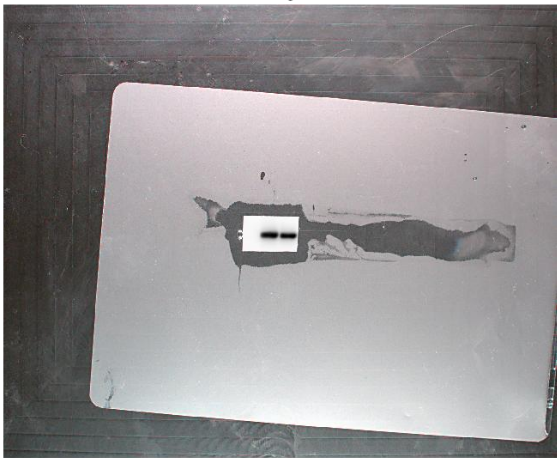

Supplement: Supplementary file 1 — Supplementary Material 1 [file 12885_2023_10903_MOESM1_ESM.pdf]
